# Controlling Cyanobacterial Blooms in Hypertrophic Lake Taihu, China: Will Nitrogen Reductions Cause Replacement of Non-N_2_ Fixing by N_2_ Fixing Taxa?

**DOI:** 10.1371/journal.pone.0113123

**Published:** 2014-11-18

**Authors:** Hans W. Paerl, Hai Xu, Nathan S. Hall, Guangwei Zhu, Boqiang Qin, Yali Wu, Karen L. Rossignol, Linghan Dong, Mark J. McCarthy, Alan R. Joyner

**Affiliations:** 1 Institute of Marine Sciences, The University of North Carolina at Chapel Hill, Morehead City, North Carolina, United States of America; 2 State Key Laboratory of Lake Science and Environment, Nanjing Institute of Geography & Limnology, Chinese Academy of Sciences, Nanjing, PR China; 3 Marine Science Institute, The University of Texas at Austin, Port Aransas, Texas, United States of America; University of New South Wales, Australia

## Abstract

Excessive anthropogenic nitrogen (N) and phosphorus (P) inputs have caused an alarming increase in harmful cyanobacterial blooms, threatening sustainability of lakes and reservoirs worldwide. Hypertrophic Lake Taihu, China’s third largest freshwater lake, typifies this predicament, with toxic blooms of the non-N_2_ fixing cyanobacteria *Microcystis* spp. dominating from spring through fall. Previous studies indicate N and P reductions are needed to reduce bloom magnitude and duration. However, N reductions may encourage replacement of non-N_2_ fixing with N_2_ fixing cyanobacteria. This potentially counterproductive scenario was evaluated using replicate, large (1000 L), in-lake mesocosms during summer bloom periods. N+P additions led to maximum phytoplankton production. Phosphorus enrichment, which promoted N limitation, resulted in increases in N_2_ fixing taxa (*Anabaena* spp.), but it did not lead to significant replacement of non-N_2_ fixing with N_2_ fixing cyanobacteria, and N_2_ fixation rates remained ecologically insignificant. Furthermore, P enrichment failed to increase phytoplankton production relative to controls, indicating that N was the most limiting nutrient throughout this period. We propose that *Microcystis* spp. and other non-N_2_ fixing genera can maintain dominance in this shallow, highly turbid, nutrient-enriched lake by outcompeting N_2_ fixing taxa for existing sources of N and P stored and cycled in the lake. To bring Taihu and other hypertrophic systems below the bloom threshold, *both* N and P reductions will be needed until the legacy of high N and P loading and sediment nutrient storage in these systems is depleted. At that point, a more exclusive focus on P reductions may be feasible.

## Introduction

Excessive phosphorus (P) and nitrogen (N) inputs promote hypertrophic conditions in lakes worldwide [1], [2], often manifested as harmful (toxic, hypoxia-generating, food-web disrupting) cyanobacterial blooms [3], [4]. Some cyanobacteria can convert atmospheric nitrogen (N_2_) to biologically-available ammonia via N_2_ fixation, providing diazotrophic cyanobacteria with an advantage in waters that are replete in other essential nutrients (e.g., P, Fe, trace metals) but deficient in N [5]. This observation forms a basis for the paradigm that low N:P ratios favor cyanobacterial dominance [6], [7]. However, N_2_ fixation is energetically unfavorable, and cyanobacteria relying on atmospheric N_2_ often do not achieve maximal growth rates [8].

Nutrient loading dynamics have changed, however, since this paradigm was first introduced [6], [7]. Agricultural, urban, and industrial expansion in developed and developing regions has dramatically increased reactive N discharges [9], while P loading has stabilized or decreased after recognition that P enrichment influenced eutrophication in freshwater ecosystems [c.f., 10]. Today, unprecedented amounts of biologically-available N are discharged over land, through subsurface aquifers, and via the atmosphere [9]. Freshwater and marine ecosystems are now N:P enriched and more eutrophic [11], [12]. Recent studies have shown that these ecosystems can become even more eutrophic when they receive persistent N loading (i.e., they remain sensitive to N inputs) [1], [2], [13].

The increasing dominance and geographic expansion of non-N_2_ fixing cyanobacterial genera, specifically blooms of the widespread, toxin-producing genus *Microcystis*, are a troubling indicator of excessive N loading [14]. This pattern has been observed in large lake systems (e.g., Lake Erie, North American Great Lakes; Lake Kasumigaura, Japan; Lake Okeechobee, Florida; Lake Taihu, China), and estuarine and coastal waters (e.g., Baltic Sea, Europe; Potomac River-Chesapeake Bay and the San Francisco Bay Delta, North America; Peel Harvey and Swan River Estuaries, Australia) [15]. *Microcystis* spp., and some benthic analogues (e.g., non-N_2_ fixing *Lyngbya* and *Oscillatoria*), are indicative of N-over-enriched conditions [16]. The continuing threat from N-driven eutrophication persists because: 1) some N can be ‘lost’ by denitrification, creating a demand for continued N inputs to sustain eutrophication [17]; 2) historically high P loads accumulate in sediments, providing a reservoir for recycled P to sustain eutrophication; and 3) in nutrient-affected eutrophic lakes, N_2_ fixation does not meet ecosystem-scale N requirements [12], [18], [19]. These studies indicate that eutrophication is accelerated by increasing external N inputs, and they point to the importance of controlling N inputs (in addition to P) for reducing and reversing eutrophication along the freshwater to marine continuum [2], [20].

Schindler et al. [21] and others [22], [23] suggest that N_2_ fixing cyanobacteria will replace non-N_2_ fixers once N inputs are reduced, especially if P remains available. This possibility is troubling in lakes and reservoirs used for drinking water, fishing, and recreational purposes, because some N_2_ fixers (e.g., *Anabaena* spp., *Aphanizomenon* spp., *Nodularia* spp.) also produce toxins. We evaluated this potential scenario in highly eutrophic (hypertrophic) Lake Taihu, China’s third largest freshwater lake, which has experienced massive cyanobacterial blooms dominated by *Microcystis* spp. [24]–[26]. Previous in situ bioassay work [26], [27] in Lake Taihu has shown that P inputs control algal production in the winter-spring, but N-limitation and N and P co-limitation are common during the summer-fall bloom period. Blooms in Lake Taihu remain dominated by non-N_2_ fixing *Microcystis*, with potentially N_2_ fixing *Anabaena* and *Aphanizomenon* present as sub-dominant genera. The blooms are initiated in late spring (April–May) and persist well into fall (November).

Using in situ 1000 L mesocosms deployed for several weeks during the peak bloom period (June-August), we examined the response of the native phytoplankton community to a range of nutrient enrichments, including one in which only P was administered to enhance N-limitation. The aim of these experiments was to assess the potential for N_2_ fixing cyanobacteria to replace non-N_2_ fixers under N limited conditions in Lake Taihu.

## Methods

### Location and field sites

**Figure 1:**
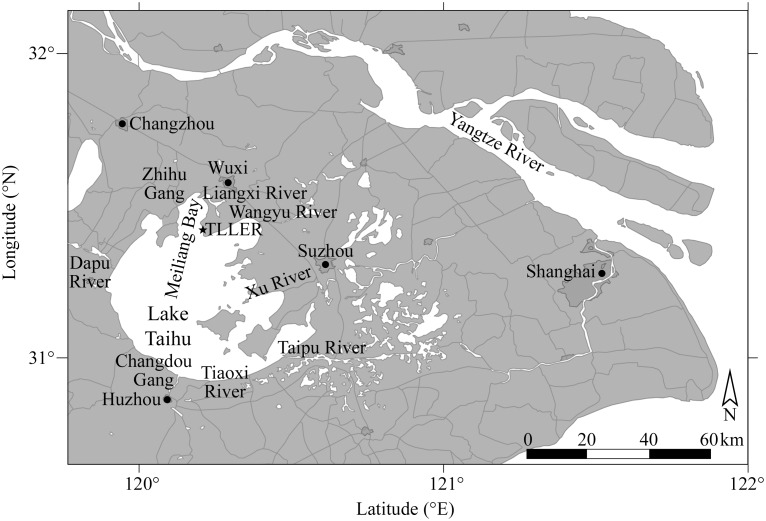
Map of Lake Taihu showing major tributaries and nearby cities. The location of Taihu in China is shown on the inserted map.

Lake Taihu (Taihu means ‘large lake’ in Mandarin) is located approximately 150 km west of Shanghai, China (Lake coordinates 31° 10′ N, 120° 9′ E). Taihu is China’s third largest freshwater lake at ∼2400 km^2^ (Fig. 1). It is shallow (mean depth ∼2 m), polymictic, and hypertrophic, and it was formed as a former oxbow of the nearby Yangtze River [28]. Taihu’s drainage basin is 36,500 km^2^ and contains more than 30 input sources, ranging from rivers to small streams and man-made drainage canals. Water exits the southeastern corner of Taihu via the Taipu River, which drains through Shanghai into the East China Sea (Fig. 1). About 40 million people live within Taihu’s watershed, which supports approximately 11% (and expanding) of the Chinese economy [28]. The lake is the key drinking water source for ∼10 million people, but also serves as a repository for waste from the urban, agricultural, and industrial segments of the local area.

No specific permissions were required for field sampling and studies. These studies did not involve endangered or protected species.

Mesocosm bioassays were located at the interface of one of the northern bays, Meiliang Bay, and the lake proper (Fig. 1). This area was chosen because it is the site of recurring, intensive annual *Microcystis* spp. blooms [24], [28], previous nutrient limitation studies [27], and is located near the Nanjing Institute of Geography and Limnology’s Taihu Laboratory for Lake Ecosystem Research (TLLER) at Wuxi. Meiliang Bay receives freshwater inputs from the Liangxi and Zhihu Gang rivers, which drain partially-treated wastewater from industrial, residential, and agricultural areas. The named rivers in Fig. 1 contribute more than 85% of the lake’s freshwater inflow.

### Mesocosms

**Figure 2:**
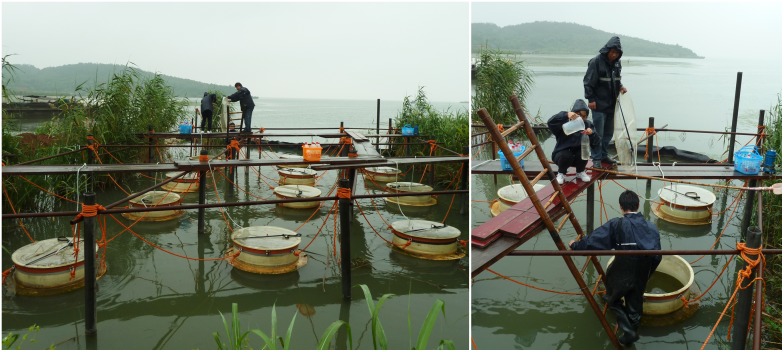
Photographs of mesocosm array, located on the shore of Taihu near the Taihu Laboratory for Lake Ecosystem Research (TLLER), at Wuxi, China.

Twelve cylindrical, 1 m diameter, 1.8 m tall, 1000 L volume, semi-translucent fiberglass mesocosms, which are open at the top and closed at the bottom, were constructed by the shipyard in Wuxi. The mesocosms were installed at the lakeshore near TLLER (Fig. 2), providing ambient natural light and temperature conditions. The mesocosms were outfitted with a flotation flange to ensure that the mesocosms would remain upright and above the lake surface water line. We also monitored daily ambient physical and chemical parameters (temperature, transparency, dissolved oxygen, pH, conductivity), nutrient concentrations (N, P, and carbon species), and phytoplankton community biomass and composition in the mesocosms and nearby lake water. The mesocosm experiment was started on 15 July and ended on 31 July 2013.

We measured phytoplankton community biomass and compositional response to N limitation (i.e., exclusive P enrichment) vs. P limitation (N enrichment), as well as co-limitation, over the two-week period, which allowed the phytoplankton community to adjust to selective nutrient enrichment over numerous generations. The potential for a shift from non-N_2_ fixing to actively N_2_ fixing cyanobacteria is supported by previous (and ongoing) microscopic observations showing that both groups co-existed during N replete and N-limited periods [24], [27].

Prior to deployment, the mesocosms were cleaned and ‘conditioned’ by filling several times with water from Meiliang Bay (Fig. 1). Once filled for the experiments, the mesocosms were allowed to stabilize for 24 h prior to initiating the following triplicated treatments: 1) Control (no nutrients); 2) nitrogen only (N-only); 3) phosphorus only (P-only); and 4) nitrogen and phosphorus addition (N+P). Nitrogen was added as a solution of 0.3 mg L^−1^ NO_3_-N (as KNO_3_) and 0.2 mg L^−1^ NH_4_-N (as NH_4_Cl; final concentration 0.5 mg L^−1^ N). Phosphorus was added as 0.02 mg L^−1^ P (as K_2_HPO_4_). These nutrient enrichments were based on recent (since 2007) nutrient measurements during spring in Meiliang Bay prior to the bloom initiation [26], [27]. Nitrogen and P were supplied every two days throughout the experiment.

While we recognize the importance of sediments as a source or sink for nutrients, we did not add sediments to the mesocosm experiment because: 1) the objective of this study was to evaluate the potential for planktonic N_2_ fixers to dominate under well-defined water column N and P regimes; 2) preliminary experiments (in 2012) indicated that the presence or absence of sediments had little effect on phytoplankton community structure and N_2_ fixation rates over the course of the mesocosm deployments; and 3) we were concerned that sediments would affect DIN availability via denitrification and excessive microbial (non-phytoplankton) uptake, which could introduce variability, lead to poor replication, and defeat the purpose of the experiment (examining water column utilization of nutrients).

Mesocosms were thoroughly stirred with a plastic paddle following nutrient additions and daily prior to sampling to maintain homogeneous nutrient and seston distributions. Growth of periphyton on the mesocosm walls was controlled by scrubbing the walls of mesocosms every two days with a pre-cleaned brush. This procedure proved effective in minimizing periphyton growth during the experimental period. Subsamples were collected every two days for nutrient analyses (dissolved inorganic N and P forms, Total N and P), carbon and N content of the seston and chlorophyll *a* (Chl *a*)). Samples were filtered within 2 h of collection on 25 mm Whatman GF/F filters (pre-combusted at 450°C for elemental C and N analyses). Retained material was used for particulate analyses and filtrate was analyzed for dissolved nutrients. Subsamples for photopigments (fucoxanthin, 9′cis-neoxanthin, violaxanthin, diadinoxanthin, antheraxanthin, myxoxanthophyll, alloxanthin, lutein, zeaxanthin, chlorophyll *b*, *ß*-carotene, echinenone), which are diagnostic of major phytoplankton taxonomic groups, N_2_ fixation potential, and microscopic identification and enumeration of phytoplankton were collected on GF/F filters every four days. All subsamples were collected prior to nutrient additions. Water temperature, dissolved oxygen, and pH were measured with a YSI Model 6600 multiprobe.

### Nutrient and other chemical analyses

Soluble reactive P (SRP) was determined using the molybdenum blue method [29]. NH_4_^+^-N was measured by the indophenol blue method, and NO_x_ (NO_3_^–^-N + NO_2_^−^ N) was analyzed with the cadmium reduction method [29]. Total phosphorus (TP), total dissolved phosphorus (TDP), total nitrogen (TN), and total dissolved nitrogen (TDN) were analyzed using a combined persulphate digestion [30], followed by spectrophotometric analysis, as for SRP and NO_3_^–^-N.

### Photopigment determinations

Chlorophyll *a* served as an indicator of total phytoplankton biomass, while diagnostic chlorophyll and carotenoid photopigments were used as indicators of major phytoplankton classes [31]. For Chl *a*, 50–100 mL subsamples were collected from mesocosms and filtered onto 25 mm GF/F filters, which were blotted dry, folded in foil wrappers and frozen until analysis. Chl *a* concentrations were determined spectrophotometrically after extraction in 90% hot ethanol [32].

Diagnostic photopigments were measured using high performance liquid chromatography (HPLC). Approximately 100–150 mL of sample water was vacuum filtered using 25 mm GF/F filters (nominal pore size 0.7 µm). Filters were blotted dry, frozen and transported (frozen) to The University of North Carolina at Chapel Hill, Institute of Marine Sciences for analysis. Filters were extracted in 100% acetone, sonicated, and stored at 20°C for approximately 24 h. Extracts (200 µL) were then injected (using an autosampler) into a Shimadzu Model SIL-20AC HT HPLC equipped with a Model SPD-M10Avp photo diode array detector, following procedures described by Van Heukelem et al. [33] and Pinckney et al. [31]. Pigments were identified according to their absorption spectra, which were determined using commercially obtained pigment standards (DHI-Danish Hydraulic Institute, Denmark).

Contributions of the dominant four algal classes (diatoms, chlorophytes, cryptophytes, and cyanobacteria) to Chl *a* were calculated using Chemtax [34]. The input pigment ratio matrix for the four classes was adapted from Schlüter et al.’s [35] study of lacustrine phytoplankton species. Average ratios for each class were calculated based on species averages within their light availability experiments. With only four dominant classes identified microscopically, the number of pigments used for Chemtax was reduced to twelve. Dominant photopigments, algal classes represented, and input ratios used for the Chemtax analysis are shown in Table 1. Phytoplankton samples were fixed with Lugol’s iodine solution (2% final concentration) and sedimented for 48 h. Cell density was measured with a Sedgwick–Rafter counting chamber under microscopic magnification of X200–400. Phytoplankton species were identified according to Hu et al. [36].

**Table 1:** Dominant algal classes and input accessory pigment ratios used for Chemtax determination of class contribution to total chlorophyll *a*.

	Fuco	Neo	Viol	Diad	Anth	Myx	Allo	Lut	Zea	Chl *b*	*β* Car	Ech
Diatom	0.51	0	0	0.074	0	0	0	0	0	0	0.003	0
Cryptophyte	0	0	0	0	0	0	0.37	0	0	0	0.004	0
Chlorophyte	0	0.038	0.026	0	0.016	0	0	0.15	0	0.36	0.003	0
Cyanobacteria	0	0	0	0	0	0.14	0	0	0.28	0	0.097	0.076

Fuco = fucoxanthin; Neo = 9′cis-neoxanthin, Viol = violaxanthin; Diad = diadinoxanthin; Anth = antheraxanthin; Myx = myxoxanthophyll; Allo = alloxanthin; Lut = lutein; Zea = zeaxanthin; Chl *b* = chlorophyll b; *β* Car = *β*-carotene; Ech = echinenone.

### Nitrogen fixation

Nitrogen fixation (nitrogenase activity) rates were estimated using the acetylene reduction (AR) assay, as described by Paerl [37], by dispensing 90 mL of sample into 125 mL stoppered serum vials, and adding 7 mL of acetylene generated from calcium carbide (Aldrich). Triplicate light and dark bottles, as well as 0.2 µm filtered (sterile PALL Inc. nitrocellulose filters) lake water blanks were incubated in situ in Taihu for 4 h, after which 4 mL headspace samples were collected in pre-evacuated Vacutainer (Becton Dickinson Inc.) tubes. The tubes were then transported to the laboratory, and 0.2 mL was withdrawn for measurements of ethylene production (from acetylene) by flame ionization gas chromatography using a Shimadzu GC9 gas chromatograph.

### Statistical analyses

Phytoplankton responses to the factorial nutrient addition design were assessed using an unsupervised, objective, nutrient-limitation classification model [38]. The model is based on a generalized linear model of treatment contrasts and time effects accounted for by orthogonal polynomials. Aikake information criteria (AIC) was used to determine the model that achieves the greatest degree of parsimony with biomass responses to treatments and time. Separating the time effects from the treatment effects provides a classification of which nutrient, if any, was limiting, or whether both nutrients were limiting. Seven classifications of nutrient limitation status are possible: 1*-null*, meaning no nutrient effects; 2 *and* 3*- exclusive N or P limitation*, where only N or P stimulates biomass above the control, and addition of the non-limiting nutrient in the N+P treatment provides no additional stimulation; 4 and 5*-primary N or P limitation*, where addition of one nutrient stimulates growth above the control, but addition of the non-limiting nutrient provides additional stimulation; 6*-combined limitation*, where both N, P, and N+P additions are different from the control; and 7*-exclusive combined limitation*, when neither N nor P additions alone stimulate biomass above the control, but the combined N+P treatment stimulates growth. This classification model was used to analyze responses of total phytoplankton biomass, as Chl *a*, and the biomass responses of specific taxa based on diagnostic photopigments or microscopic enumeration. To determine confidence in each nutrient limitation classification, bootstrapping with replacement was used to produce 1000 resampled data sets from the original triplicated mesocosm treatments. The frequency of bootstrapped nutrient limitation determinations provides a measure of confidence in the determination made from the original experimental dataset [38].

## Results

### Nutrient addition effects on phytoplankton biomass

**Figure 3:**
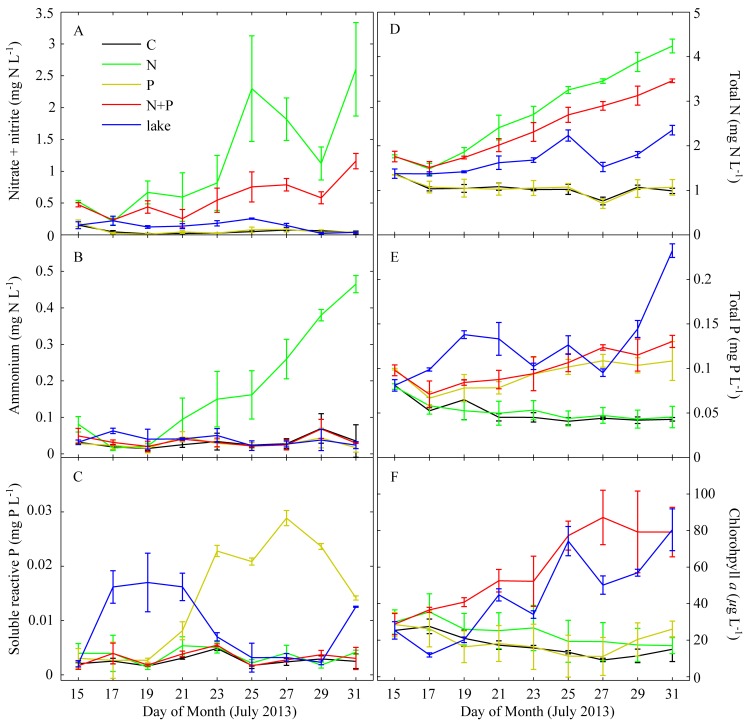
Time series of dissolved and total nitrogen and phosphorous forms and phytoplankton biomass as chlorophyll *a* from the summer 2013 mesocosm experiment. Solid lines connect means of triplicate mesocosms. Error bars are one standard deviation.

For the first two days, N-only additions led to a small but significant stimulation of phytoplankton biomass relative to controls and P additions (Fig. 3). Similarity between N-only and N + P additions showed that the initial community was N limited rather than co-limited. After two days, only the N+P treatment showed continued growth, with Chl *a* concentrations reaching 60 µg L^−1^ or higher, reflecting typical bloom conditions [24]–[26]. All other treatments showed similar declines in Chl *a* through 27 July 2013.

Overnight on 27 July into early morning 28 July 2013, the TLLER meteorological station recorded 48.6 mm rainfall during a strong thunderstorm. Nutrient concentrations (in mg L^−1^ N or P) in the rainwater were 3.41 TN, 3.39 TDN, 0.46 NH_4_^+^, 0.69 NO_3_^−^, 0.032 TP, 0.20 TDP, and 0.025 PO_4_^3−^. If only inorganic nutrients are assumed bioavailable, the N and P added to the 1000 L mesocosms would have increased bioavailable N and P concentrations by ∼0.04 and 0.0009 mg L^−1^ (102∶1 molar ratio), respectively. If all N and P added from the rain were assumed bioavailable, N and P bioavailability would have increased by 0.12 and 0.0012 mg L^−1^ (221∶1 molar ratio), respectively. This N enriched and P poor rain event served as a ‘natural experiment’ to evaluate the effects of N addition to the experimentally-manipulated mesocosm phytoplankton communities. The atmospheric N addition led to a rapid stimulation of biomass in the P addition treatments and, to a lesser extent, in the controls. N-only and N + P treatments showed no response, indicating that growth in those treatments was not N limited during the time of the storm. Assessments of nutrient limitation status by the unsupervised nutrient limitation classification model were affected by the storm and corroborate the overall N limitation status of the phytoplankton community. For the entire experimental period, the nutrient limitation model best supported by the data was a primary combined N and P limitation (Table 2), but 23% of the resampled datasets supported a primary N limitation determination. When only data prior to the storm were analyzed, confidence in a primary N limitation status for Chl *a* response was high (93% of resampled datasets), with a small portion (6.3%) of primary combined limitation determinations (Table 2).

**Table 2:** Nutrient limitation classifications (N.L.C.) and ambiguity of classification for total phytoplankton biomass and specific algal taxa (Andersen et al. 2005).

Group	N.L.C.	Null	XN	N1	XP	P1	XC	C1
Total	C1	0	0	22.5	0	3.3	1.1	73.1
phytoplankton	(N1)	(0)	(0)	(93.3)	(0)	(0.2)	(0.2)	(6.3)
Diatoms	P1	0	0	1.3	0	51.4	1.6	45.7
	(XC)	(0)	(0)	(20.0)	(0)	(9.9)	(45.5)	(24.6)
Cryptophytes	N1	0	0	54.2	0	18.7	11.5	15.6
	(N1)	(0)	(0)	(64.2)	(0)	(5.2)	(25.3)	(5.3)
Chlorophytes	C1	0	0	31.9	0	0	8.5	59.6
	(N1)	(0)	(0)	(9.7)	(0)	(3.2)	(7.9)	(79.2)
Cyanobacteria	N1	0	0	38.4	0	30.6	12.1	18.9
	(N1)	(0)	(0)	(40.5)	(0)	(29.5)	(9.5)	(20.5)
*Microcystis* sp.	P1	0	0	1.4	0	87.7	0.3	10.6
	(P1)	(0)	(12.0)	(2.8)	(0)	(69.1)	(0.5)	(15.6)
*Anabaena* spp.	–	7.8	35.7	18.1	11.5	28.0	1.5	13.7
	(–)	(11.3)	(64.0)	(7.7)	(0)	(5.7)	(9.2)	(2.1)

Total phytoplankton biomass as spectrophotometric Chl *a*. Ambiguity of the classification of the nutrient limitation status is shown by the frequency (%) of different classifications from 1000 bootstrapped data sets. Values in parentheses are results excluding data from the period following the 27 July 2013 rain event. A – indicates that the null model was chosen in greater than 5% of boot-strapped cases.

### Phytoplankton community comparisons between mesocosms and ambient lake water

**Figure 4:**
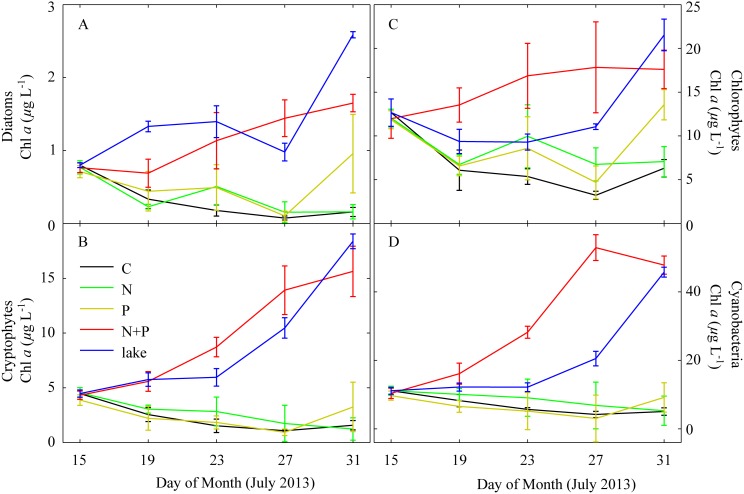
Time series of biomass of the dominant algal classes from the summer 2013 mesocosm experiment. Solid lines connect means of triplicate mesocosm tanks. Error bars are one standard deviation.

When examined over time for phytoplankton biomass and composition, ambient lake water tended to show elevated levels of total community biomass and major algal groups relative to the control (no nutrient addition) mesocosms (Fig. 4). These differences were observed shortly after the initiation and remained throughout the duration of the experiment (16 d), indicating that nutrient-limited conditions were maintained in the controls throughout the experiment. While total biomass (as Chl *a*) was consistently lower in controls, the phytoplankton composition remained similar between controls and ambient lake water, indicating that potentially confounding issues, such as selective grazing, death, or major shifts in phytoplankton composition due to ‘container effects’ were minimal. While phytoplankton biomass remained low in control mesocosms, Chl *a* and other diagnostic photopigment concentrations remained detectable throughout the experiment (Fig. 4).

### Nutrient addition effects on phytoplankton community composition

Overall, the addition of either N or P alone showed little stimulation of the dominant phytoplankton classes over the control; however, combined N+P additions stimulated all four major classes (Fig. 4). For diatoms, P additions led to small increases in biomass above the control. The nutrient classification status was assessed as primary P limitation, but confidence in this determination was weak with primary combined limitation being nearly as likely (Table 2). Exclusion of the post-storm periods revealed that, prior to this point, the diatom community had likely been co-limited (Table 2). Cryptophytes showed weak stimulation by N-only additions (Fig. 4) and were classified as having a primary N limitation status. However, confidence in this assessment was not strong with primary P limitation, exclusive N + P co-limitation, and primary combined limitation being collectively as likely (Table 2). Chlorophytes were assessed as primary combined limitation, and exclusion of the post-storm period increased confidence in this assessment. Cyanobacteria were assessed as primarily being N limited but, as with the other phytoplankton classes, confidence in this determination was weak. It is instructive that for none of the four phytoplankton classes, in any of the 1000 bootstrapped data sets, was there a single instance where nutrient limitation status was assessed as exclusive N or exclusive P limitation. This underscores the finding that the dominant phytoplankton classes were largely co-limited by N and P during the experiment. N and P availability nearly balanced demand such that, when N or P was added alone, it drove growth limitation to limitation by the other nutrient. In this case, only combined additions of N and P led to continued accumulation of biomass.

The lack of cyanobacterial biomass stimulation by P additions was surprising, since ambient phosphate concentrations were quite low at the start of the experiment (∼2–4 µg P-PO_4 _L^−1^). Total dissolved inorganic N (DIN) (NH_4_^+^ + NO_x_) concentrations in the P treatments were also quite low (∼20–40 µg DIN L^−1^), providing presumably favorable conditions (DIN:DIP of approximately 10) for selective stimulation of cyanobacterial growth, especially among N_2_ fixing genera in response to P additions [7].

**Figure 5:**
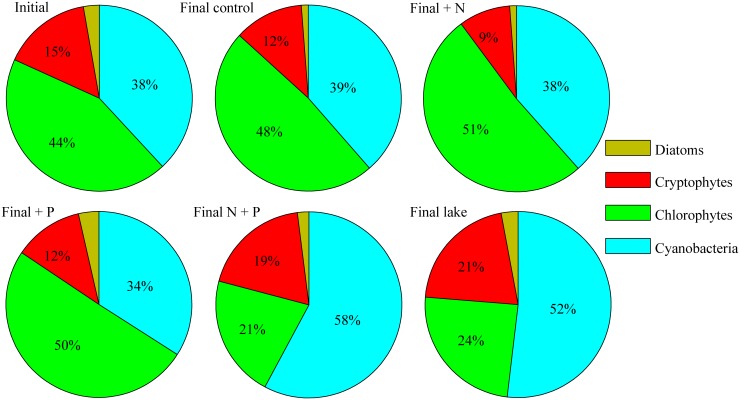
Pie graphs depicting proportion of total chlorophyll *a* comprised by each of the dominant four algal classes at the initial sampling of the mesocosm experiments (average of all samples collected) and final sampling (average of triplicate mesocosms) for each treatment and the ambient lake water.

N+P additions promoted the growth of all groups relative to controls, with cyanobacteria showing particularly strong stimulation (Fig. 3). This appeared to mirror conditions in ambient lake water, which showed a steady stimulation of cyanobacterial biomass and bloom development in parallel with the experimental period. By the end of the experiment, community composition of the N+P treatments were very similar to ambient lake water (Fig. 5). In comparison, community composition of the controls, P-only, and N-only treatments was similar to the initial community composition (Fig. 5).

### Nutrient conditions during the mesocosm experiment

Dissolved inorganic and total N and P concentration showed strong patterns related to nutrient additions and biological utilization (Fig. 3). Control and P only addition mesocosms showed almost no detectable DIN, indicating that N was likely the most limiting nutrient throughout the experimental period. Even with N additions, for the first two and four days, NOx and NH_4_^+^ concentrations decreased in both the N-only and N+P addition treatments. This indicates that N availability did not meet N demand at the beginning of the experiment and suggests that NH_4_^+^ was preferentially utilized over NO_x_. After this initial period, both NO_x_ and NH_4_^+^ increased in the N-only additions. However, the N+P additions treatments showed half the NO_x_ buildup and no significant NH_4_ accumulation, indicating that while N limitation was more evident than P limitation, N+P co-limitation most likely characterized conditions during the experimental period. Total N predictably increased in response to N-only and N+P enrichment, but notably it did not increase in response to P-only enrichment. This served as independent (of acetylene reduction measurements) evidence that N_2_ fixation did not respond in a significant manner to the P-only treatments and that microbial production in response to the P-only treatments was likely supported on regenerated sources of N. SRP remained at low levels similar to the control in the N-only and N+P treatments. In the P-only treatment, SRP accumulated after four days and reached a peak on 27 July. After the 27 July storm, SRP dropped by 50%, indicating significant uptake in response to increased N availability from rainwater. Total P increased in response to both P-only and N+P additions, but there were no significant differences in response to these treatments. Overall, nutrient concentration results support previous findings that during summer months this region of Taihu is N+P co-limited [26], [27]. Exclusive P limitation was not detected throughout the course of these experiments.

### Microscopic observations of phytoplankton community dynamics

**Figure 6:**
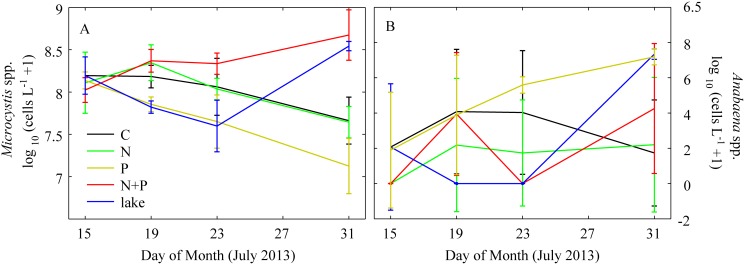
Time series of cell abundances for *Microcystis* spp. and *Anabaena* spp., the dominant non-nitrogen fixing and nitrogen fixing genera during the summer 2013 mesocosm experiment. Solid lines connect means of log_10_ values of triplicate mesocosm tanks. Error bars are one standard deviation.

**Figure 7:**
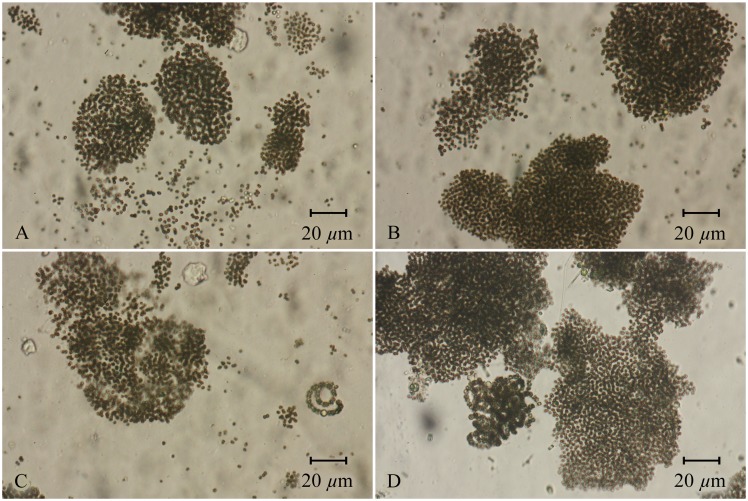
Representative microscopic observations (X200, brightfield) of dominant cyanobacterial genera in the mesocosm experiment conducted during 2013. Samples were collected towards at the end of the experiment, 31 July 2013. (A) Control (no nutrient addition). (B) N-only addition. (C) P-only addition. (D) N+P addition. Note the overall dominance by *Microcystis* spp. colonies. However, an increase in number of filaments of the N_2_ fixing genus *Anabaena* was observed in the phosphorus and phosphorus and nitrogen treatments. No significant N_2_ fixation was observed in either of these treatments however. Biomass-wise, *Microcystis* spp. clearly dominated the cyanobacterial community in all treatments.

In general, microscopic observations tended to confirm HPLC diagnostic pigment analyses with respect to the responses of major phytoplankton groups to various nutrient additions. Specific attention was paid to the dominant N_2_ fixing (*Anabaena* sp.) vs. non-N_2_ fixing (*Microcystis* sp.) cyanobacterial genera responses to various nutrient additions (Figs. 6, 7).

The diatom community was dominated by a mix of solitary centric (e.g., *Cyclotella* sp.), pennate (e.g., *Navicula* sp.), and chain-forming genera (e.g., *Melosira* sp.). Chlorophytes proved to be highly diverse throughout all treatments, but the largest fraction of chlorophyte biomass was comprised of colonial (e.g., *Scenedesmus* spp., *Crucigenia* sp., *Pediastrum* spp., *Eudorina* sp.) and filamentous (*Ulothrix* sp.) forms. Cryptophytes were largely comprised of nanoplanktonic *Cryptomonas* spp. and *Chroomonas* spp. In general, dominant members of these eukaryotic groups were similar to those reported during a long-term monitoring study [24]. Dominant cyanobacterial genera in order of prominence were *Microcystis*, *Oscillatoria*, *Anabaena*, *Aphanizomenon*, and *Merismopedia*.

Among these, *Anabaena* and *Aphanizomenon* are capable of N_2_ fixation. *Microcystis* responded in two ways to the nutrient addition treatments: stimulation by N+P additions, and a negative response to P-only additions (Fig. 6). The nutrient limitation classification for *Microcystis* was assessed as primary P limitation because the nutrient classification does not differentiate between positive or negative effects [38]. For more than 8% of the resampled datasets, a null model fit the data equally as parsimoniously as a model that included treatment contrasts for cell abundances of *Anabaena*. This is conceptually equivalent to an insignificant *F*-value in an ANOVA and indicates that high variability within replicates and a high frequency of zero counts precluded an objective assessment of its nutrient limitation status. However, by the end of the experiment, *Anabaena* had highest cell densities in the P-only treatments (Fig. 6). *Aphanizomenon* abundance was also highly variable but showed declines in abundance for all treatments.

### Nitrogen fixation responses

**Figure 8:**
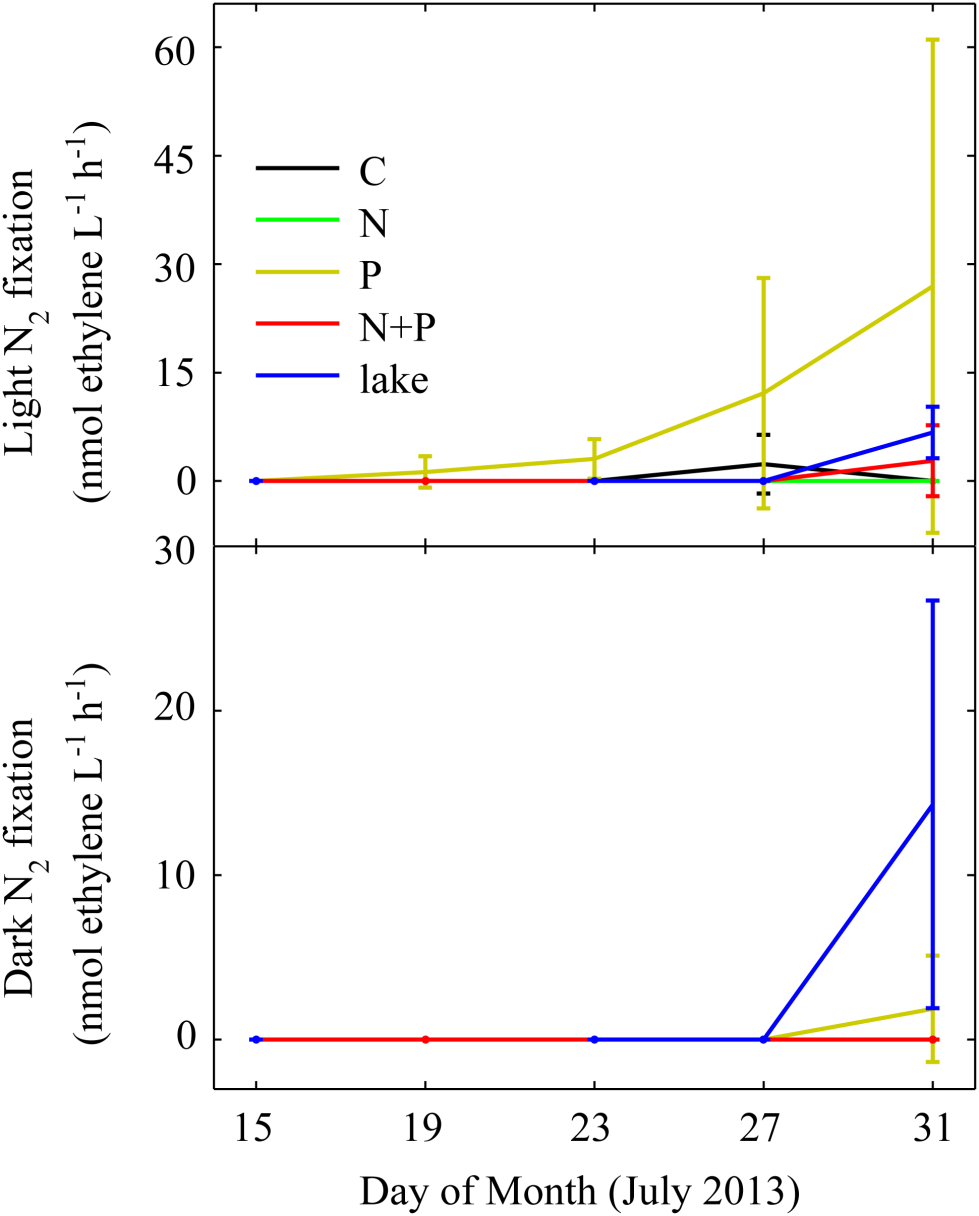
Time series of light and dark acetylene reduction rates from the summer 2013 mesocosm experiment. Solid lines connect means of triplicate mesocosms. Error bars are one standard deviation.

Nitrogen fixation (as acetylene reduction) responses were monitored throughout the experimental period. Extremely low (and statistically insignificant) rates of N_2_ fixation were observed relative to filtered lake water and reagent blanks in both the lake and among mesocosm treatments (Fig. 8). These results support the above-mentioned microscopic observations indicating that, while filamentous N_2_ fixing genera *Anabaena* and *Aphanizomenon* were present, and *Anabaena* increased in the P-only treatment (Fig. 6), heterocyst frequency was very low, and only traces of N_2_ fixation were observed at the end of the experiment (Figs. 7, 8). Therefore, while the potential for this process exists, there was no significant activity. These results were supported by direct measurements of total N (TN) in the mesocosms, which showed no evidence that N_2_ fixation led to a significant increase in TN in response to P additions. This proved true for all treatments, including the P-only treatment, which was expected to yield the highest potential for N_2_ fixation.

We calculated the percentage of *Anabaena’s* N requirement (based on growth rates and cell N quotas) that may have been satisfied by N_2_ fixation, based on our AR measurements and conversions to N_2_ fixed using a 4∶1 ethylene production:N_2_ fixation ratio. At most, only 20% of *Anabaena’s* N needs were met this way. If we extrapolate these results to the entire phytoplankton community, less than 5% of N needs are met by N_2_ fixation under P-enriched conditions. There were no significant rates of N_2_ fixation in any of the other treatments.

These results indicate that despite the presence of N limited and N+P co-limited conditions, diazotrophic genera must be competing with non-N_2_ fixing genera (i.e., *Microcystis* and eukaryotes) for combined N sources to sustain growth. This situation persisted throughout the experimental period.

Interestingly, the P only treatments did not stimulate total cyanobacterial community production. Instead, only diatoms showed selective stimulation by this treatment, and even then, this stimulation only occurred early in the experiment. Overall, enhanced N limitation (through selective P enrichment) did not lead to a significant increase in cyanobacterial biomass or result in N_2_ fixation, but the N+P treatment did enhance cyanobacterial biomass (Fig. 4).

## Discussion

Results from the mesocosm nutrient addition experiment confirm prior work based on short-term (<1 week) microcosms, indicating that, during summer bloom periods, growth of Taihu’s cyanobacteria-dominated phytoplankton community is primarily controlled by N supply and further stimulated by N and P together [26], [27]. Despite summer N limitation, N_2_ fixing cyanobacteria did not significantly increase in abundance, heterocyst frequency, or N_2_ fixing activity, either in the mesocosm experiments or in ambient lake water. These results do not support Schindler et al.’s [21] conclusion that diazotrophic cyanobacteria should dominate and quantitatively fix N_2_ to compensate for N requirements needed to sustain phytoplankton community production rates. The length of mesocosm experiments of up to a month should have provided plenty of time for numerous generations of N_2_ fixers to proliferate and for vegetative cells to differentiate into heterocysts [5], [8]. Hence, the low N fixation rates were not attributable to short incubation time. The question therefore arises: Why were non-N_2_ fixing genera (*Microcystis*) not replaced by active diazotrophs (*Anabaena, Aphanizomenon*) during P enrichment, despite the fact that these genera were observed in the lake throughout the experimental period?

In addressing this question, we note that phytoplankton growth also may be constrained by other factors, including temperature, availability of micronutrients (Fe, trace metals), and adequate light for supporting energy-demanding processes, such as N_2_ fixation. Other ecological factors could play additional roles, including the ability to regulate buoyancy (important for accessing light and nutrients), grazing, and beneficial as well as antagonistic associations with other microbes. These factors are known to play a role in the competitive interactions between diazotrophic and non-diazotrophic cyanobacterial species [3].

In shallow, well mixed, highly turbid Taihu, the ability of non-diazotrophs to access and store nutrients, while maintaining high photosynthetic rates and competing for N sources with diazotrophs, is likely important in the initiation and maintenance of blooms. The non-diazotroph *Microcystis* dominated prior to the experiment and remained dominant while N was severely limiting (i.e., P alone treatment). N limitation constrained the ability of a bloom, equal in biomass to the ambient lake, to form. *Microcystis* maintained its dominance over N_2_ fixing genera under N limitation and during a decrease in overall biomass. Its ability to effectively compete for dwindling N sources, especially NH_4_^+^ regenerated from sediments and organic N, provides an advantage [39], [40]. *Microcystis* dominance may also be related to: 1) effective cellular N storage; 2) access to sediment and water column regenerated N (as NH_4_^+^) via buoyancy regulation; 3) close associations with heterotrophic bacteria present around colonies, which enhanced ‘phycosphere’-scale nutrient (including N and P) recycling [41]; and 4) more efficient use of light for supporting photosynthetic growth and nutrient sequestration than diazotrophic genera. The latter selective mechanism may be quite important in Taihu’s turbid, shallow waters, in which *Microcystis* may be particularly effective in vertically orienting itself [42].

Since N_2_ fixation is a very energy-demanding process [5], light availability in highly turbid Taihu may be insufficient for diazotrophs to compete with the effective N sequestration mechanisms that *Microcystis* possesses [39], [43]. Secchi depths in Taihu are commonly in the range of 8–85 cm (mean = 38 cm; [44], [45], which indicates that a substantial amount of the 2 m deep water column experiences very low light levels (<5% of incident light). Suspended solids, and not chlorophyll or chromophoric organic matter, accounts for the vast majority of light attenuation in Taihu [45]. Therefore, we suggest that light limitation may interact with nutrient limitation in this hypertrophic lake to favor non-N_2_ fixing cyanobacteria, especially during maximum bloom periods, when surface scums effectively shade the underlying water column. Other shallow, highly turbid lakes (e.g., Lake Okeechobee, FL., Dutch lakes) show similar patterns, with *Microcystis* maintaining dominance over N_2_-fixing genera in N and light limited conditions [46], [47].

Iron (Fe) limitation could have played a role in the inability of N_2_ fixers to assume dominance under the extreme N-limited conditions imposed in these experiments [5]. However, recent examinations indicated Fe-replete conditions during the summer blooms in Taihu [48]. Therefore, this is not a likely explanation for the absence of this process.

From a nutrient management perspective, results from our mesocosm experiments indicate that more N limited conditions induced by selectively enrichment with P did not lead to replacement of non-diazotrophs with diazotrophs in Taihu. This scenario may become more probable as overall nutrient reductions, leaching of stored nutrient supplies in sediments, and increases in transparency take place over time. However, this scenario will likely take several years to decades to emerge in this large, relatively long residence time (∼1 year) lake. Therefore, we conclude that N input reductions, in concert with P reduction (i.e., a dual nutrient strategy), is the most effective near-term strategy for reducing phytoplankton biomass, bloom potentials, and improving the overall trophic state of Taihu, as well as downstream N limited Yangtze estuary and South China Sea coastal waters. This approach requires a specific set of nutrient reduction targets, which are being formulated through the combined use of nutrient addition and dilution bioassays [26], [27]. At present, it appears that N input reductions would not lead to replacement of *Microcystis* with N_2_ fixers, even though this outcome could be considered a positive outcome, given the tendency for lower toxicity, growth rates, and ultimate biomasses of N_2_ fixers [8].

The use of microcosm and mesocosm experiments for determining phytoplankton responses to nutrient inputs has been criticized by Carpenter [49] as not reflective of ecosystem-level conditions. These approaches employ small-scale containers over short (days to weeks) incubation times and may not adequately mimic temporal and spatial scales for nutrient cycling and fluxes. However, prior work has shown remarkable agreement among nutrient enrichment and limitation experiments carried out over these time intervals with a wide size range of incubation vessels [50], [51]. Furthermore, incubations of at least two weeks should be adequate to elicit taxa-specific (including diazotrophic) responses to nutrient enrichment and other ecophysiological factors (e.g., changes in irradiance, temperature, pO_2_) [52].

We previously examined this question in Lake Taihu using 1 L to 4 L polyethylene Cubitainers over a time span of 1 week or less [26], [27]. These experiments indicated that N and P co-limitation was prevalent, especially during the critical summer bloom period. Others [51], [53] have shown similar results in eutrophic to hypereutrophic lakes. The question persists, however; can these results be extrapolated over longer time scales, and do they reflect natural conditions, such as sediment-water column nutrient exchange and seasonally-variable physical (temperature. light), chemical, and biological (grazing, microbial interactions) conditions? Can species and group succession patterns be replicated over the relatively short time scales that these experiments are conducted? This question is particularly relevant to the issue of replacement of non-N_2_ fixing with N_2_ fixing cyanobacterial species.

In shallow, hypertrophic Lake Taihu, there was remarkable agreement between ambient lake water and mesocosms deployed in the lake. As N limitation was intensified during the summer period, the non-N_2_ fixing cyanobacterial community was not replaced by a N_2_ fixing community. Most likely, *Microcystis* remained dominant during P enriched conditions in the lake because it effectively competes for regenerated N (as NH_4_^+^) from sediments and the water column [27], [54], as noted in previous mesocosm experiments in a shallow, eutrophic, Swedish lake [39]. Energetically, *Microcystis* must be able to access regenerated N more efficiently than *Anabaena* and *Aphanizomenon* can fix N_2_ to meet their N requirements. The tradeoffs of regenerated N vs. fixed N need further study, especially relative to selective nutrient management strategies aimed at bringing hypertrophic lakes, like Taihu, below bloom thresholds. Our results, combined with the fact that sediment N:P is low in Taihu [55] and most eutrophic lakes, show that *both* N and P reductions are needed in the short-term, in large part due to the legacy of high N and P loading and sediment nutrient storage common in this and other eutrophic lakes [7], [47], [56]. For this reason, reducing only P loading to Lake Taihu, without parallel N reductions, is unlikely to improve water quality (i.e., reductions in bloom frequency and intensity) on a time scale that will be acceptable to the public and managers.

Recent evidence suggests that N loading may indirectly cause P release from sediments via disruption of the sulfur and Fe cycles [57], providing additional justification for a dual nutrient management approach. Failure to control N loading also has consequences for aquatic systems located downstream [20]. Excessive N loads to coastal and estuarine systems is linked to widespread development of algal blooms, such as red tide, and bottom-water hypoxia (e.g., the northern Gulf of Mexico). A P-only approach to watershed nutrient management for Taihu and other freshwater systems simply shunts N-driven eutrophication issues further downstream to the N sensitive coastal zone [20], an approach that we believe is irresponsible.
